# Supplementary material for: Development of a Noninfectious Japanese Encephalitis Virus Replicon for Antiviral Drug Screening and Gene Function Studies
Source: Viruses. 2025 May 27;17(6):759. doi: 10.3390/v17060759 (PMC12197453; doi:10.3390/v17060759)
Supplement: Supplementary file 1 [file viruses-17-00759-s001.zip › Supplementary Fig. 1.pptx]

## Slide 1
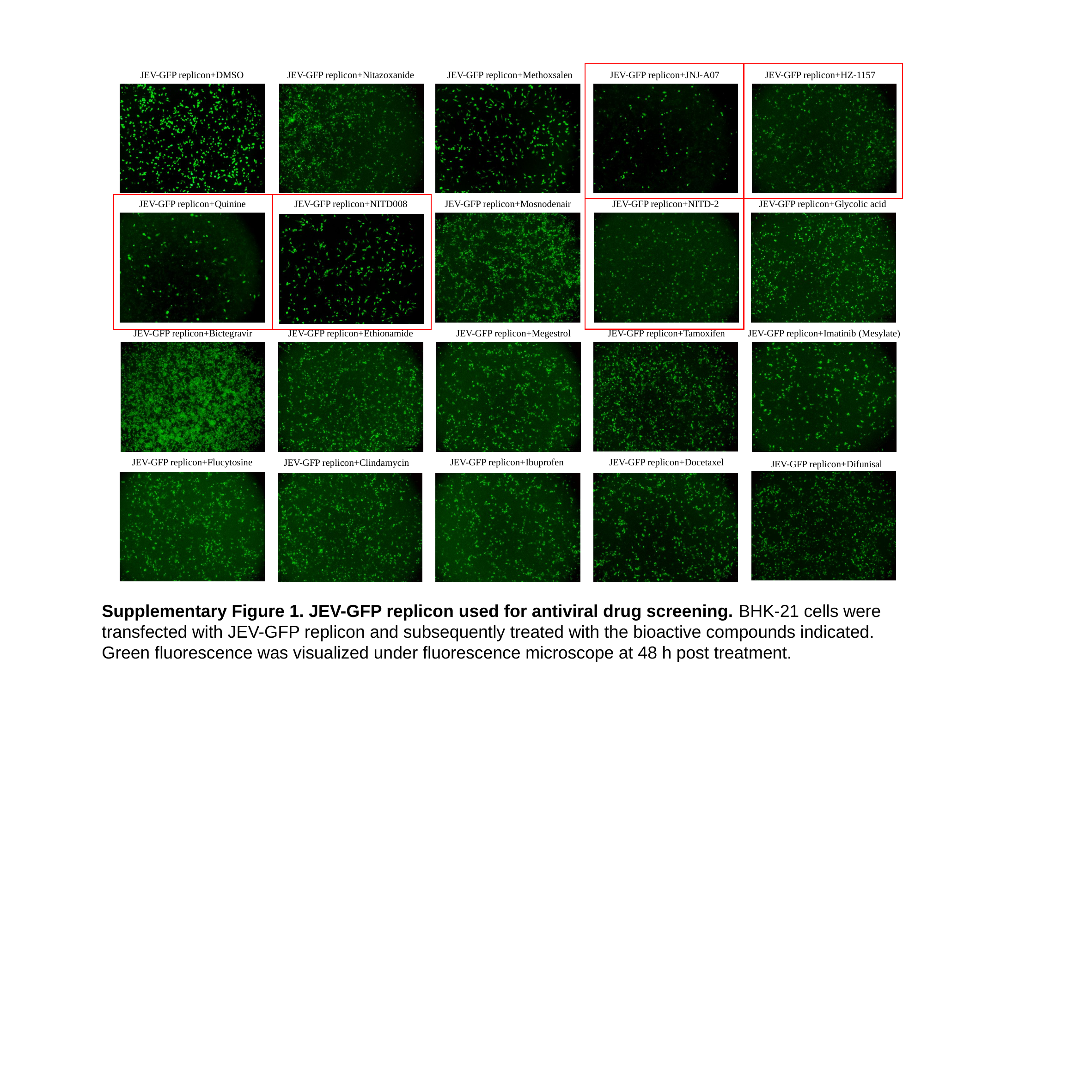

JEV-GFP replicon+Nitazoxanide
JEV-GFP replicon+Methoxsalen
JEV-GFP replicon+HZ-1157
JEV-GFP replicon+DMSO
JEV-GFP replicon+JNJ-A07
JEV-GFP replicon+Quinine
JEV-GFP replicon+NITD008
JEV-GFP replicon+Mosnodenair
JEV-GFP replicon+NITD-2
JEV-GFP replicon+Glycolic acid
JEV-GFP replicon+Bictegravir
JEV-GFP replicon+Ethionamide
JEV-GFP replicon+Megestrol
JEV-GFP replicon+Tamoxifen
JEV-GFP replicon+Imatinib (Mesylate)
JEV-GFP replicon+Flucytosine
JEV-GFP replicon+Ibuprofen
JEV-GFP replicon+Docetaxel
JEV-GFP replicon+Clindamycin
JEV-GFP replicon+Difunisal
Supplementary Figure 1. JEV-GFP replicon used for antiviral drug screening. BHK-21 cells were transfected with JEV-GFP replicon and subsequently treated with the bioactive compounds indicated. Green fluorescence was visualized under fluorescence microscope at 48 h post treatment.
