# Supplementary material for: Development of a Noninfectious Japanese Encephalitis Virus Replicon for Antiviral Drug Screening and Gene Function Studies
Source: Viruses. 2025 May 27;17(6):759. doi: 10.3390/v17060759 (PMC12197453; doi:10.3390/v17060759)
Supplement: Supplementary file 1 [file viruses-17-00759-s001.zip › Supplementary Fig. 2.pptx]

## Slide 1
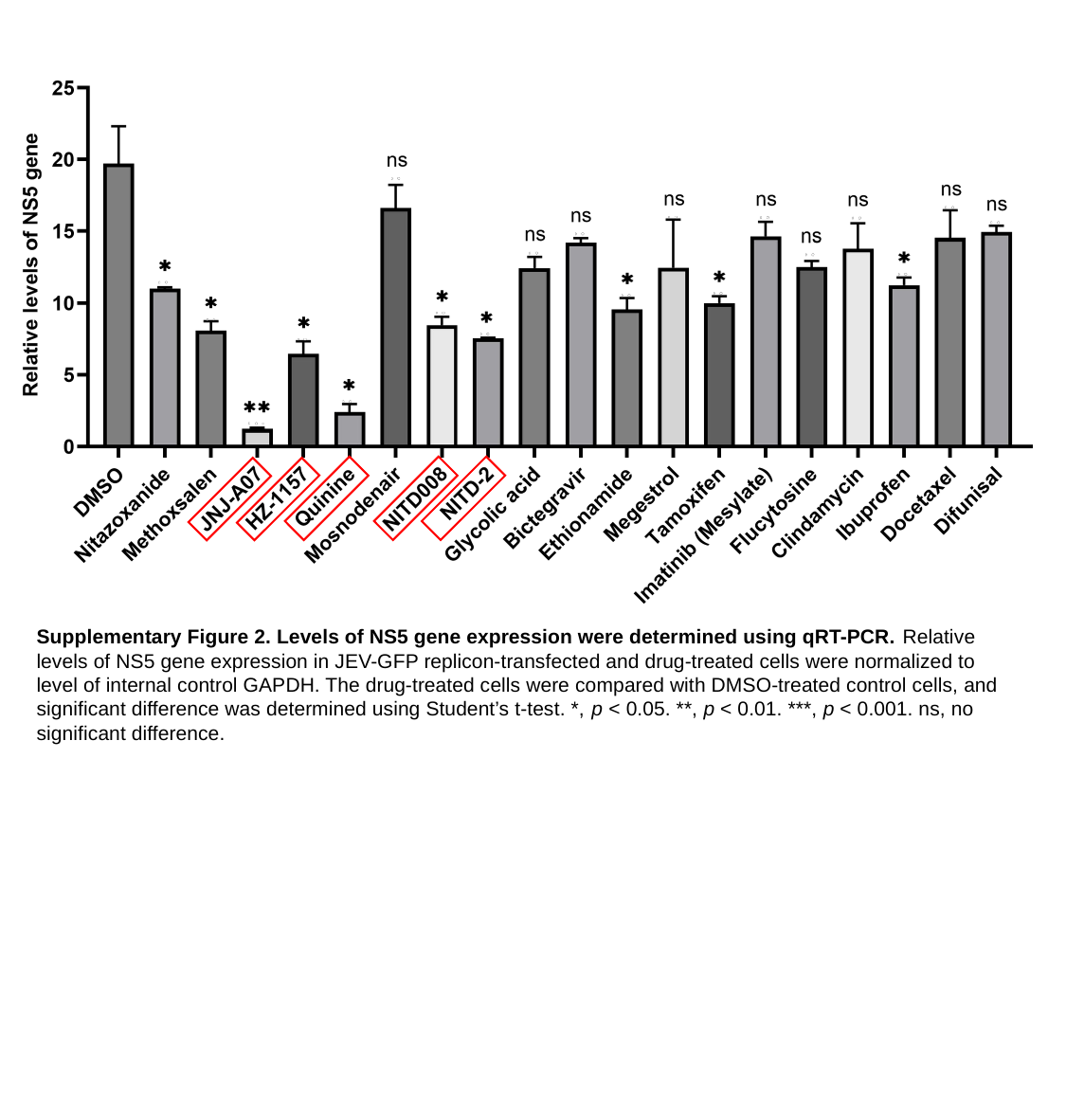

Supplementary Figure 2. Levels of NS5 gene expression were determined using qRT-PCR. Relative levels of NS5 gene expression in JEV-GFP replicon-transfected and drug-treated cells were normalized to level of internal control GAPDH. The drug-treated cells were compared with DMSO-treated control cells, and significant difference was determined using Student’s t-test. *, p < 0.05. **, p < 0.01. ***, p < 0.001. ns, no significant difference.
