# Supplementary material for: Development of a Noninfectious Japanese Encephalitis Virus Replicon for Antiviral Drug Screening and Gene Function Studies
Source: Viruses. 2025 May 27;17(6):759. doi: 10.3390/v17060759 (PMC12197453; doi:10.3390/v17060759)
Supplement: Supplementary file 1 [file viruses-17-00759-s001.zip › Supplementary Table 1.pdf]

Supplementary Table 1 Primers used in this study

Primer Name	Primer Sequence (5'-3')
JEV-F-1	tacccggtaccgtcggatccagaagtttatctgtgtgaac
JEV-R-1	tccagcctgcttcagcaggctgaagttagtagctccgcttccaactaccc
JEV-F-2	gaagtctgctaacatgcggtgacgtcgaggagaatcctggcccatcaatcgccctggct
JEV-R-2	ggaaagggatcagtggtcccgggcggggtcgccgtcataa
JEV-F-3	ttatgacggcgaccccgcccgggaccactgatccctttcc
JEV-R-3	gggaccatgccggcctctagaagatcctgtgttcttcctca
GFP-F	ctgaagcaggctggagacgtggaggagaaccctggacctgtgagcaagggcgaggagct
GFP-R	accgcatgttagcagacttcctctgccctctccactgcccttgtacagctcgtc
JEV C4A-R	gtcaatggcagcgccagtgtcggcatgcacattggtcgct
JEV C4A-F	gcgaccaatgtgcatgccgacactggcgctgccattgac
NS3-qPCR-F	gttcgtggcaagtgtgaaaa
NS3-qPCR-R	gatgtcagtggtgataaca
NS1-qPCR-F	tgtggaagtggcatcttcg
NS1-qPCR-R	actcccacatctggtgttc
