# Supplementary material for: Development of a Noninfectious Japanese Encephalitis Virus Replicon for Antiviral Drug Screening and Gene Function Studies
Source: Viruses. 2025 May 27;17(6):759. doi: 10.3390/v17060759 (PMC12197453; doi:10.3390/v17060759)
Supplement: Supplementary file 1 [file viruses-17-00759-s001.zip › Supplementary Table 2.pdf]

Supplementary Table 2 Information of the bioactive compounds used in this study

Drug name	Purity	Stock solution	Storage concentration	Storage temperature
Nitazoxanide	99.95%	DMSO	10mM	-80 ℃
Methoxsalen	99.98%	DMSO	10mM	-80 ℃
JNJ-A07	≥ 98.0%	DMSO	10mM	-80 ℃
HZ-1157	98.16%	DMSO	10mM	-80 ℃
Quinine	98.05%	DMSO	10mM	-80 ℃
NITD008	98.00%	DMSO	10mM	-80 ℃
Mosnodenair	99.31%	DMSO	10mM	-80 ℃
NITD-2	99.36%	DMSO	10mM	-80 ℃
Glycolic acid	≥97.0%	DMSO	10mM	-80 ℃
Bictegravir	99.79%	DMSO	10mM	-80 ℃
Ethionamide	99.73%	DMSO	10mM	-80 ℃
Megestrol	99.44%	DMSO	10mM	-80 ℃
Tamoxifen	99.78%	DMSO	10mM	-80 ℃
Imatinib (Mesylate)	≥ 98%	DMSO	10mM	-80 ℃
Flucytosine	99.99%	DMSO	10mM	-80 ℃
Clindamycin	99.90%	DMSO	10mM	-80 ℃
Ibuprofen	99.97%	DMSO	10mM	-80 ℃
Docetaxel	99.94%	DMSO	10mM	-80 ℃
Difunisal	99.93%	DMSO	10mM	-80 ℃
